# Preliminary User-Centred Evaluation of a Bio-Cooperative Robotic Platform for Cognitive Rehabilitation in Parkinson’s Disease and Mild Cognitive Impairment: Insights from a Focus Group and Living Lab in the OPERA Project

**DOI:** 10.3390/jcm14197042

**Published:** 2025-10-05

**Authors:** Ylenia Crocetto, Simona Abagnale, Giulia Martinelli, Sara Della Bella, Eleonora Pavan, Cristiana Rondoni, Alfonso Voscarelli, Marco Pirini, Francesco Scotto di Luzio, Loredana Zollo, Giulio Cicarelli, Cristina Polito, Anna Estraneo

**Affiliations:** 1 IRCCS Fondazione Don Carlo Gnocchi, 50143 Florence, Italy; ycrocetto@dongnocchi.it (Y.C.); sabagnale@dongnocchi.it (S.A.); gmartinelli@dongnocchi.it (G.M.); sdellabella@dongnocchi.it (S.D.B.); epavan@dongnocchi.it (E.P.); crpolito@dongnocchi.it (C.P.); 2 Research Unit of Advanced Robotics and Human-Centred Technologies, Università Campus Bio-Medico di Roma, 00128 Rome, Italy; cristiana.rondoni@unicampus.it (C.R.); f.scottodiluzio@unicampus.it (F.S.d.L.); l.zollo@unicampus.it (L.Z.); 3 Khymeia Group, 35129 Padua, Italy; a.voscarelli@khymeia.com (A.V.); m.pirini@khymeia.com (M.P.); 4 UOC Neurologia, AORN S.G. Moscati, 83100 Avellino, Italy; cicalio@libero.it

**Keywords:** bio-cooperative device, focus group, living lab, multimodal platform, Parkinson’s disease, robot-aided rehabilitation

## Abstract

**Background:** Mild cognitive impairment (MCI) affects up to 40% of patients with Parkinson’s disease (PD), yet conventional rehabilitation often lacks engagement. The OPERA project developed a novel Bio-cooperative Robotic Platform (PRoBio), integrating a service robot and a virtual reality-based rehabilitation for personalized cognitive training. This work presents two preliminary user-centred studies aimed to assess PRoBio usability and acceptability. **Methods:** to gather qualitative feedback on robotic and virtual reality technologies, through ad hoc questionnaires, developed according to participatory design principles and user-centered evaluation literature, Study 1 (Focus group) involved 23 participants: 10 PD patients (F = 6; mean age = 68.9 ± 8.2 years), 5 caregivers (F = 3; mean age = 49.0 ± 15.5), 8 healthcare professionals (F = 6; mean age = 40.0 ± 12.0). Study 2 (Living Lab) tested the final version of PRoBio platform with 6 healthy volunteers (F = 3; mean age = 50.3 ± 11.0) and 8 rehabilitation professionals (F = 3; mean age = 32.8 ± 9.9), assessing usability and acceptability through validated questionnaires. **Results:** The focus group revealed common priorities across the three groups, including ease of use, emotional engagement, and personalization of exercises. Living Lab unveiled PRoBio as user-friendly, with high usability, hedonic quality, technology acceptance and low workload. No significant differences were found between groups, except for minor concerns on system responsiveness. **Discussion:** These preliminary findings support the feasibility, usability, and emotional appeal of PRoBio as a cognitive rehabilitation tool. The positive convergence among the groups suggests its potential for clinical integration. **Conclusions:** These preliminary results support the feasibility and user-centred design of the PRoBio platform for cognitive rehabilitation in PD. The upcoming usability evaluation in a pilot study with patients will provide critical insights into its suitability for clinical implementation and guide further development.

## 1. Introduction

**Figure 1:**
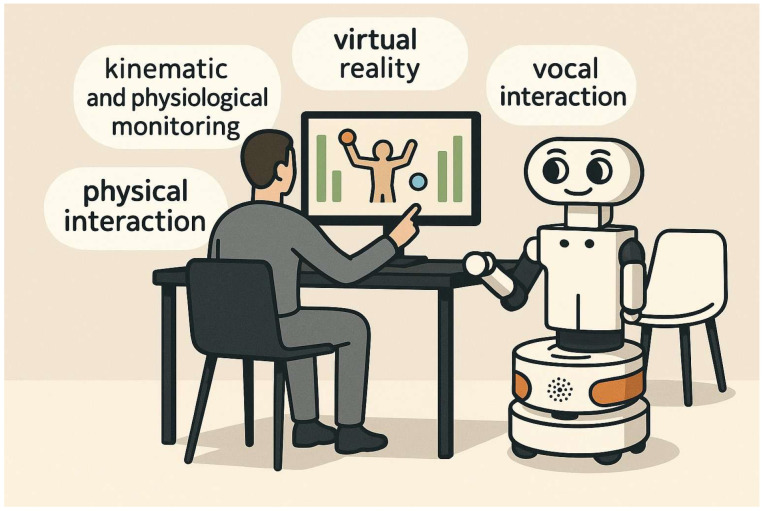
Overview of the PRoBio bio-cooperative platform.

Parkinson’s disease (PD) is a progressive neurodegenerative disorder characterized by predominant motor symptoms such as resting tremor, rigidity, and bradykinesia, typically affecting one side of the body in the early stages [1]. Alongside these motor impairments, PD is frequently associated with a broad spectrum of non-motor symptoms, among which mild cognitive impairment (MCI) is prevalent and impactful. PD-MCI is marked by impairments of multiple cognitive domains, including attention, working memory, executive functioning, visuospatial skills, and language [2,3]. Epidemiological studies estimate that approximately 40% of people with PD exhibit MCI, with rates increasing up to 75% over the disease course [4,5]. PD-MCI is widely recognized as a prodromal stage of PD dementia [6]. MCI in PD negatively impacts patients’ autonomy, daily functioning, and adherence to rehabilitation [7]. A recent Cochrane review provided preliminary evidence supporting the feasibility and potential benefits of cognitive training in improving global cognition in individuals with PD-MCI or PD dementia [8]. However, the Cochrane review also highlighted methodological limitations and heterogeneity among studies and underscored the need to explore more engaging and adaptable tools such as virtual reality (VR). Over the past decade, VR has emerged as a promising technology for the rehabilitation of PD [9], as this computer-based technology enables users to interact with simulated environments that replicate real-life scenarios [10]. Depending on the level of immersion, VR systems can be classified as non-immersive, semi-immersive, or fully immersive. Non-immersive VR typically involves interaction through standard screens or interfaces, semi-immersive systems employ large displays or projections, and fully immersive systems rely on head-mounted displays to provide a heightened sense of presence. All VR formats share the ability to engage users in controlled, task-oriented environments, which is particularly beneficial in neurorehabilitation where engagement and realism are crucial for therapeutic success [9,11]. Within these settings, patients can practice functional movements, cognitive tasks, and daily-life activities in a safe and motivating context, enhancing skill acquisition and recovery. In this context, the OPERA Project (Integrated biO-cooPErative Robotic plAtform for virtual cognitive–motor training in Parkinson’s Disease) aims to develop and evaluate the usability of an innovative Bio-Cooperative Robotic Platform (PRoBio, Figure 1), conceived for personalized VR-based cognitive rehabilitation of patients with PD-MCI. The PRoBio will be composed of a modular VR system (VRRS Compact, Khymeia SRL, Padua, Italy) and a service robot (TIAGo, PAL Robotics SL, Barcelona, Spain), to further facilitate a more interactive and adaptive rehabilitation process [12] (see Figure 1). The mobile robot is equipped with a 7-DOF anthropomorphic arm, an RGB-D camera (depth and colour) embedded in a pan-tilt head, an adjustable-height torso, and a mobile base. The robot also includes microphones and speakers for vocal interaction, and an end-effector with a two-finger gripper (i.e., robotic arm). The VRRS Compact is a non-immersive VR module with a LCD touchscreen interface, designed to deliver customizable motor and cognitive exercises. As part of the new ProBio platform, the VRRS will provide standardized cognitive tasks, targeting executive functions, memory and attention, with real-time visual and auditory feedback to enhance performance monitoring and user engagement. Simultaneously, the TIAGo robot will enable participants to interact with the VR system through its robotic arm, which functions as a joystick-like input interface for the cognitive training exercises. It also will provide verbal feedback during rehabilitation. Integrated into the biocooperative PRoBio platform, the robot will incorporate wearable sensors (Zephyr BioHarness^TM^, Medtronic/Zephyr Technology and Shimmer GSR+ module, Shimmer Sensing, Dublin, Ireland) to record heart and respiratory rates and galvanic skin response. Together with RGB-D camera and sensors, the platform will monitor the user’s biomechanical (e.g., posture), and psychophysiological state (e.g., arousal and emotional valence). This will allow adaptation of task difficulty and feedback, supporting highly personalized and engaging interventions [12,13]. Within the OPERA project a pilot, single-arm open-label clinical study will assess for the first time the clinical usability and ability of the final version of PRoBio platform in a cohort of patients with PD-MCI. Here, we present the results of two user-centred studies.

Study 1, a focus group, was carried out with patients with PD, caregivers, and healthcare professionals before the platform was developed. The aim of Study 1 was to assess perceptions and expectations regarding the use of robotic and virtual reality in cognitive rehabilitation, to guide system design and ensure alignment with the real needs of future users.

Study 2 applied a Living Lab approach to evaluate a preliminary version of the ProBio platform, specifically the integration of the VRRS module and TIAGo robot, in a realistic clinical setting with healthcare providers and healthy volunteers. The focus was on usability and clinical workflow (protocol management and user interaction), while the bio-cooperative components (e.g., wearable sensor-based monitoring) were excluded from this phase and are being assessed separately in a different cohort of healthy participants. These features, along with patient and therapist usability, will be explored further in the upcoming pilot study involving individuals with PD-MCI.

## 2. Study 1: Focus Group

### 2.1. Rationale

A focus group is a qualitative research method commonly used to explore perceptions, beliefs, needs, and attitudes of a specific target population through guided group discussions [14]. It is particularly valuable in the early stages of system design or intervention development, where it allows researchers to collect rich, detailed insights into users’ experiences and preferences. In user-centred design, the focus group evaluate usability, acceptability, and perceived value of new technologies, facilitating iterative refinement prior to large-scale deployment. Drawing on established focus group procedures, Study 1 adopted a hybrid method: following a targeted presentation delivered during a patient-centered conference (i.e., the National Parkinson’s Disease Day conference, at the AORN Moscati Hospital, Avellino, Italy) participants were invited to complete structured ad hoc questionnaires and engage in informal, individual and general discussion with the research team. This format allowed for the collection of both qualitative and quantitative feedback, preserving the participatory and exploratory nature of focus group methodology while adapting it to the practical context of the event.

### 2.2. Methods

#### 2.2.1. Participants

A sample of 23 individuals participated in the study: 10 patients with PD (F = 6; mean age = 68.9 years, SD = 8.2), 5 family members (all primary caregivers; F = 3; mean age = 49.0 years, SD = 15.5), and 8 healthcare professionals (F = 6; mean age = 40.0 years, SD = 12.0; medical doctors = 3, psychologists = 2, physiotherapists = 2, and nurse = 1). No a priori sample size calculation was performed, as focus groups aim to collect qualitative insights rather than statistical representativeness [14]. Inclusion criteria were a diagnosis of PD (for the patient group), being a primary caregiver of a PD patient, or being a healthcare professional with clinical experience in PD. Exclusion criteria included severe cognitive decline or psychiatric disorders that could limit compilation of questionnaires.

Most patients had been diagnosed with PD for over 4 years, and more than half had a disease duration exceeding 6 years. All patients were receiving pharmacological treatment at the time of the study. In addition, 7 out of 10 were undergoing physiotherapy, 3 were attending speech therapy sessions, and 2 reported participating in complementary physical activities such as gym-based rehabilitation.

Caregivers reported being involved in various aspects of patient care (e.g., toileting, medication administration, support with daily activities), closely reflecting the disease duration reported by their loved ones. As with the patients, many caregivers (4 out of 5) reported having provided support for more than 6 years. Healthcare professionals reported an extensive clinical experience with PD, with most of them indicating more than 6 years of work with PD patients, and only one professional reporting less than 3 years of experience. This background suggested a high level of familiarity with both early and advanced stages of the disease across the professional group.

#### 2.2.2. Procedure

During the conference, the OPERA Project’s goals and structure were presented through explanatory videos illustrating the two core technologies that will be used for developing the ProBio platform (TIAGo robot and the VRSS Compact). These videos provided attendees with a clear overview of how the system’s functionality and its potential applications in cognitive rehabilitation for individuals with PD-MCI. Following the presentation, participants, particularly patients and caregivers, completed a group-administered survey designed to collect structured feedback. Afterwards, an informal exchange between the OPERA research team and the participants gathered additional qualitative impressions, concerns, and suggestions about the potential use of the two technologies in rehabilitation.

#### 2.2.3. Materials

The ad hoc questionnaire used in the Study 1 was developed by a multidisciplinary team including neuropsychologists and neurologists with expertise in PD, as well as bioengineers specialized in virtual reality and bio-cooperative robotics. The questionnaires were based on established principles from participatory design and previous literature on user-centered evaluation in digital health [15].

It was designed to assess user perceptions of the project’s goals, perceived usability, usefulness, relevance, and impact of the technological devices (and their potential combination into a unified platform) for rehabilitation. Tailored versions of the questionnaire were created for each participant group, but a common structure was maintained to ensure comparability across groups.

The questionnaire was organized into six thematic sections: i. Demographic (i.e., age, gender, caregiver relationship, if applicable, professional role for healthcare staff); ii. Clinical-Functional information of patients (i.e., duration of PD, presence of co-existing comorbidities, cognitive status, and daily habits, with specific reference to everyday situations in which patients experience the greatest difficulties, particularly those related planning, organization, or dual-tasking); iii. Technical expectations and preferences, to investigate participants preferences and expectations regarding types of exercises, level of difficulty, real-time feedback, and personalization of the rehabilitative path; iv. General health and mobility, focused on the patient’s general physical health, fatigue levels, and motor capabilities that might influence interaction with the rehabilitation system; v. Robot interaction and expectations, to investigate participants’ expectations and concerns about their potential interaction with the robot components (e.g., robotic arm, the ability of the system to recognize emotional states and fatigue); vi. Suggestions and open feedback, where participants were invited to share any additional comments, perceived advantages, or potential concerns regarding the implementation of such a system in their rehabilitation routine.

The questionnaire required approximately 10–15 min to complete. See details of questionnaire in Supplementary Materials, Section S1.

#### 2.2.4. Statistical Analysis

Descriptive statistics were computed. Chi-square tests were used to assess differences in perceptions among the three groups. The level of significance was set at *p* < 0.05 for all analyses, which were carried out with Jamovi (version 2.5, 2024) [16].

### 2.3. Results

Although most comparisons did not yield statistically significant differences between the three stakeholder groups (i.e., patients, caregivers, and healthcare professionals) across any of the investigated dimensions (*p* > 0.05), some significant effects were observed for emotional engagement ratings (*p* = 0.010) and perceived device safety (*p* = 0.019; see Table 1).

#### 2.3.1. Clinical–Functional Aspects

When asked about patients’ daily cognitive challenges, all three groups (patients, caregivers, healthcare professionals) consistently reported that following long or complex conversations was the most frequent difficulty. Spatial disorientation emerged as the second most common issue. These findings were shared across groups, with no statistically significant differences observed (*p* > 0.05). Caregivers tended to rate patients’ engagement in cognitive stimulation activities (e.g., reading, memory games) as lower than what patients self-reported, suggesting a possible discrepancy between self-perception, lived experience and external observation. However, all groups emphasized the importance of maintaining cognitive abilities, most participants rating it as “very important”.

#### 2.3.2. Technical Expectations and Preferences

Across all respondent categories, ease of use emerged as the most valued feature for the two systems (VR and service robot). Patients and caregivers also considered the ability to foster emotional engagement and enjoyment as important device features, while healthcare professionals deemed the ability to personalize the exercise and to monitor the patients as an important characteristic. All groups expressed appreciation for real-time performance feedback, which was seen as a key motivational driver. A statistically significant difference in emotional engagement ratings (*p* = 0.010) was observed, with caregivers assigning greater importance to this aspect compared to professionals. Regarding exercise preferences, patients and caregivers favored memory games, while professionals preferred simulations of daily activities (e.g., meal preparation). Across all groups, improving autonomy in daily activities was the most frequently endorsed goal of cognitive rehabilitation, followed by reducing anxiety, especially among patients and caregivers, and enhancing memory.

#### 2.3.3. General Health and Mobility

Participants reported a range of motor abilities that could affect technology interaction. Some patients and caregivers reported difficulties with tasks requiring fine motor skills (e.g., using a touchscreen or writing), although most indicated they were able to use smartphones or tablets independently. Healthcare professionals corroborated these difficulties and emphasized the need for systems designed to accommodate reduced mobility and fatigue.

#### 2.3.4. Robot Interaction and Expectations

Most participants had no previous experience with robot-assisted rehabilitation. Patients and caregivers primarily envisioned the robot as a cognitive assistant (e.g., for simulating daily tasks), while professionals also emphasized its role in providing physical guidance (e.g., for hand or arm movements) as an important characteristic of the device. The most relevant robotic applications were simulations of everyday activities (e.g., preparing meals or writing), followed by guided motor exercises. Among evaluated features, device safety was rated as significantly more important by caregivers compared to the other groups (*p* = 0.019). Across all groups, adaptability to the user’s motor ability was the most requested feature, along with movement precision and safety. While the general attitude toward the robotic arm was positive, some concerns were raised, particularly about system complexity and the possibility of users feeling “controlled” by the machine.

#### 2.3.5. Suggestions and Open Feedback

In the open-ended feedback, patients appreciated the possibility to customize rehabilitation experiences, caregivers emphasized the system’s potential to motivate and engage users, and healthcare professionals underlined the importance of tracking progress and integrating cognitive and motor training. All groups agreed that the system should combine technical functionality with emotional sensitivity, and that its design should support ease of use for non-specialist users. Several participants also emphasized the importance of emotional adaptability, highlighting the need for the system to detect stress and fatigue and adjust exercises accordingly.

## 3. Study 2: Living Lab

### 3.1. Rationale

A Living Lab is an open, user-centred innovation environment that enables testing, co-design, and validation of products or services in real-life settings through the active involvement of end-users and other stakeholders [17]. Living Labs are particularly valuable in the development of health technologies, as they provide an intermediate evaluation phase between laboratory-based prototypes and large-scale clinical implementation. They allow researchers to assess the usability, acceptability, and feasibility of a system in ecologically valid conditions, capturing realistic user behaviours, system interactions, and contextual feedback [18,19]. As such, the Living Lab acts as a crucial bridge between technological development and real-world application, enhancing system readiness and user-centred design.

In the context of the OPERA Project, the Living Lab tested the final version of the platform with a cohort of healthy volunteers and healthcare professionals in a realistic clinical setting. Patients with PD-MCI were not involved. This methodological choice was made to frame the study as a pre-clinical validation step, aimed at verifying the technical reliability, usability, and safety of the system before patient exposure. This staged approach ensured that potential technical issues could be identified and addressed without burdening vulnerable clinical populations. The ProBio usability in real-world rehabilitation context, and preliminary assessment of clinical performance will be evaluated in the pilot study on in individuals with PD-MCI.

### 3.2. Methods

#### 3.2.1. Participants

As Pallot et al. [20] pointed out, the Living Lab does not require statistical power analysis for the sample size because the main objective is to explore and optimize user interaction with the platform in a real-world context. Inclusion criteria for the participants were age of at least 18 years and no history of neurological, cognitive, or psychiatric disorders. A sample of 14 participants was enrolled at the Rehabilitation Unit of Fondazione Don Gnocchi, Sant’Angelo dei Lombardi (Italy): 6 healthy volunteers (F = 3; mean age = 50.3 years old; SD = 11.0) and 8 rehabilitation professionals (F = 3; mean age = 32.8 years; SD = 9.98; physiotherapist = 4; speech language therapists = 2; psychologist = 1; neurophysiology technician = 1). The average years of education were comparable across groups (approximately 16 years), and gender distribution did not differ significantly (χ^2^ = 0.219, *p* = 0.640).

#### 3.2.2. Procedure

**Figure 2:**
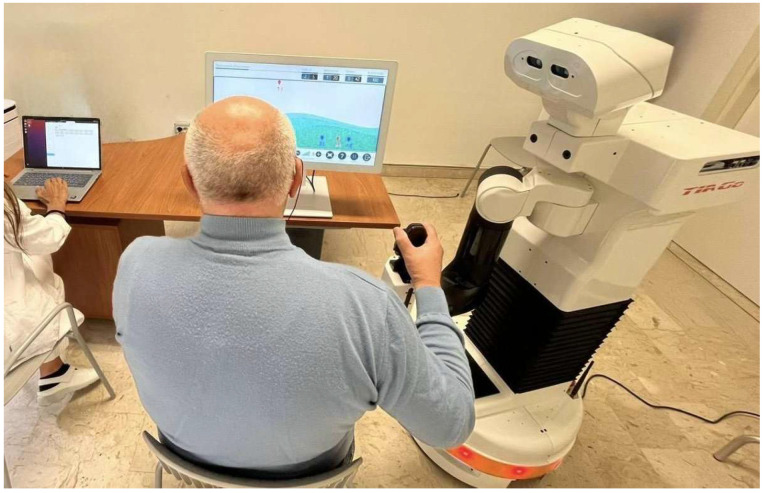
Experimental setup of the Living Lab: the robotic arm controls the VRRS exercises, while both VRRS and Robotic systems provide real-time vocal feedback to support user engagement.

Participants were engaged in a single session Living Lab, using the PRoBio platform (Figure 2). During the session, participants interacted with the PRoBio system, by performing two cognitive tasks delivered through the VRRS: one using the touchscreen and the other using the robotic arm as a joystick. The VRRS provided auditory feedback on task performance, while the robot monitored facial expressions through an RGB-D camera and delivered vocal prompts to user engagement. At the end of the testing session, each participant completed a set of standardized questionnaires designed to assess key usability-related dimensions, including ease of interaction, interface clarity, perceived usefulness of the feedback, and any difficulties encountered during use. Data collection occurred immediately after the interaction phase to ensure accurate and reliable impressions.

#### 3.2.3. Materials

##### Assessment Tools

Participants completed six standardized questionnaires assessing different dimensions of the PRoBio platform. Usability was evaluated using the System Usability Scale (SUS) [21,22] and the eHealth UsaBility Benchmarking Instrument (HUBBI) [23]. User experience, including pragmatic and hedonic quality, was measured with the short version of the User Experience Questionnaire (UEQ-S) [24]. Technology acceptance was explored using the Technology Acceptance Model (TAM) [25,26] and the Italian version of the Unified Theory of Acceptance and Use of Technology (I-UTAUT) [27]. Cognitive workload during interaction was assessed with the NASA Task Load Index (NASA-TLX) [28,29].

Detailed descriptions of each questionnaire, including structure, scoring procedures, and psychometric properties, are provided in the Supplementary Materials, Section S2.

### 3.3. Statistical Analysis

Descriptive statistics (means, standard deviations, frequencies) were computed for all questionnaires. Exploratory group comparisons were performed. All statistical analyses were performed using Jamovi software (version 2.5, 2024) [16], with the level of significance set at *p* < 0.05.

### 3.4. Results

The results obtained from the administered questionnaires organized according to the main dimensions explored are reported in Table 2.

#### 3.4.1. Usability and User Experience

Both groups rated the PRoBio as usable and user-friendly, as the SUS score showed no significant difference between professionals and volunteers (*p* = 0.236). However, healthy participants were slightly more critical on the aspects related to system confidence and consistency, as assessed by the SUS item 6 (“I found inconsistencies in the system”), and item 9 (“I felt very confident using the system”), although these differences were not statistically significant among the three groups.

This overall positive rating of the ProBio usability across the 3 groups was confirmed by the HUBBI questionnaire, which explores not only usability but also broader aspects of user experience and satisfaction. The HUBBI revealed only a significant difference in the “Basic performance and system stability” dimension (*p* = 0.041), between healthy and professional participants. This result is consistent with the SUS findings, where healthy participants also reported slightly lower scores on items related to system consistency and confidence (items 6 and 9). Healthy participants expressed more reservations about the PRoBio technical reliability than healthcare professionals. The remaining HUBBI dimensions, including interface design and presentation, navigation, clarity of information, guidance and support, showed no significant differences between the two groups. Overall, while convergence was observed across stakeholder groups regarding ease of use, personalization, and emotional engagement, some divergences also emerged. Caregivers tended to prioritize safety and motivational aspects, whereas healthy volunteers expressed concerns related to the system’s stability and technical reliability.

#### 3.4.2. Experience and Engagement

The system was perceived as both functional and engaging, as indicated by the UEQ-S questionnaire responses from both groups. Specifically, no differences emerged in pragmatic quality (related to usability and efficiency) or hedonic quality (related to enjoyment and emotional involvement), with both groups describing the platform as intuitive, stimulating, and visually appealing.

#### 3.4.3. Technology Acceptance

In line with these results, the TAM questionnaire confirmed that participants viewed PRoBio as useful and easy to use, with no significant differences in perceived usefulness or ease of use (*p* > 0.48), suggesting consistent appreciation of the platform’s potential across groups.

#### 3.4.4. Acceptance and Confidence in the System

The I-UTAUT scale further supported the positive trend, showing high ratings in terms of performance expectancy, social influence, facilitating conditions, and self-efficacy, with no significant differences between groups. Both professionals and volunteers appeared confident in their ability to use the platform, and anxiety or distrust towards the robotic component was minimal.

#### 3.4.5. Workload and Effort

Finally, the NASA-TLX results suggested that the interaction with PRoBio did not require a significant cognitive or physical workload. Both groups reported low levels of mental and physical demand, minimal frustration, and an acceptable effort-to-performance ratio (all *p* > 0.27). This indicates that the tasks and interactions were perceived as manageable and not overly stressful.

#### 3.4.6. Correlation Analysis

Correlation analyses between questionnaires total score and subscales highlighted numerous significant relationships across the questionnaires. The HUBBI scores showed multiple associations with other measures: Basic System Performance correlated with I-UTAUT total score (r = 0.55, *p* = 0.040 *), while Task-Technology Fit was positively associated with Pragmatic Quality (UEQ-S) (r = 0.55, *p* = 0.042 *), Social Influence/Empowerment (I-UTAUT) (r = 0.66, *p* = 0.010 *), and Trust (I-UTAUT) (r = 0.69, *p* = 0.006 *). Design and Interface correlated with I-UTAUT Trust (r = 0.57, *p* = 0.034*) and I-UTAUT total (r = 0.59, *p* = 0.027 *), while Navigation and Structure was related to I-UTAUT Social Influence (r = 0.54, *p* = 0.049 *) and I-UTAUT total (r = 0.59, *p* = 0.027 *). Information and Terminology was positively related to Pragmatic Quality (UEQ-S) (r = 0.61, *p* = 0.020 *) but negatively associated with NASA-TLX Frustration (r = –.77, *p* = 0.001 *). Satisfaction correlated positively with I-UTAUT Acceptance/Usefulness (r = 0.65, *p* = 0.012 *), Social Influence (r = 0.56, *p* = 0.036 *), Trust (r = 0.60, *p* = 0.023 *), and I-UTAUT total (r = 0.72, *p* = 0.004 *). The UEQ-S dimensions were strongly interrelated and connected with other questionnaires: Pragmatic Quality correlated with UEQ-S total (r = 0.92, *p* < 0.001 *). The TAM scales were also linked to workload indices: Perceived Usefulness correlated positively with NASA-TLX Physical Demand (r = 0.76, *p* = 0.002 *), while Perceived Ease of Use showed positive correlations with NASA-TLX total (r = 0.75, *p* = 0.002 *). Finally, I-UTAUT scores showed multiple significant associations: Acceptance/Usefulness correlated positively with UEQ-S total (r = 0.65, *p* = 0.012 *) but negatively with NASA-TLX Physical Demand (r = −0.72, *p* = 0.003 *). These correlation findings offer interesting insights into the relationships between usability, acceptance, and perceived workload. However, they must be interpreted with caution due to the limited sample size (*n* = 14), which restricts statistical power and increases the risk of Type I error (i.e., identifying significant associations that may have occurred by chance). Moreover, the number of comparisons performed across multiple scales and subdimensions further amplifies this risk. For this reason, the reported correlations should be considered exploratory and hypothesis-generating rather than confirmatory. No causal inferences can be drawn from these associations, and future studies with larger and more representative samples will be necessary to validate and deepen these preliminary observations.

## 4. General Discussion

The present two studies provided preliminary important information on the usability, acceptability, and perceived workload of a novel bio-cooperative platform (PRoBio), and its core components, designed for VR cognitive rehabilitation of individuals with PD-MCI.

Overall, the focus group study supported the relevance and acceptability of the PRoBio platform as a user-centred, emotionally responsive solution for cognitive rehabilitation in PD. A strong alignment was found across the three groups of participants in prioritizing specific key features of the two key components (Robot and VR system) such as ease of use, safety, exercise personalisation, and the device’s ability to adapt cognitive training to users’ emotional and physical states. This convergence across stakeholder categories supports the hypothesis that integrating these two devices in a bio-cooperative platform, like PRoBio, could effectively address the needs and expectations related to PD cognitive rehabilitation. Additionally, patients and caregivers particularly emphasized the importance of motivation and emotional engagement in the rehabilitation experience, whereas professionals were more focused on functionality and technical aspects, such as the ability to monitor the patient’s physiological and emotional state and to track performance over time. These differences offer valuable insights into the distinct expectations of each user group and can guide targeted refinements of the PRoBio system. The robotic component was generally well-received, especially for its potential in simulating everyday activities, although some concerns were expressed about its complexity and the lack of human-like interaction. Such concerns are consistent with previous literature on human–robot interaction, which highlights usability and emotional attunement as critical success factors for assistive robotic systems [30]. Participants’ desire for emotionally intelligent systems aligns with findings in “affective computing”, which indicate that user acceptance increases when technology is responsive to affective states [31]. These results underscore a shared appreciation for emotionally adaptive systems, suggesting that future iterations of the platform should incorporate real-time affective sensing to adjust task difficulty, pacing, or support strategies in accordance with the user’s emotional and physical state. Notably, limited prior experience with similar technologies among participants suggested that technological unfamiliarity may represent a potential barrier to implementation. In this context, adequate user training and gradual familiarization could be crucial for the effective adoption of PRoBio in clinical and home settings. These findings underscore the value of a co-design approach in the early validation of neurorehabilitation technologies [14]. Early integration of stakeholder perspectives enhances usability and long-term adherence [32]. The user insights gathered in Study 1 enabled the OPERA partners to guide the development of the PRoBio according to the patient’s real needs [14]. The positive outcomes of the Living Lab (Study 2), which tested the preliminary PRoBio in a semi-controlled environment with healthy volunteers and healthcare professionals, suggest that the design choices informed by the focus group feedback were appropriate and well-received. While the two studies involved different participant groups and methodologies, this alignment supports the value of incorporating user perspectives early in the development process.

Results from validated questionnaires indicated high usability (SUS), strong emotional and functional engagement (UEQ-S), and low perceived workload (NASA-TLX), as both groups reported that the platform was easy to use, efficient, and non-burdensome. In addition to usability scores, the correlation analysis revealed some noteworthy relationships. For instance, a positive correlation was observed between Perceived Usefulness and NASA-TLX Physical Demand. While this may initially appear counterintuitive, it could indicate that users who found the system more beneficial were also more engaged during the tasks, leading to a higher perception of physical effort. This suggests that usefulness may drive active involvement, even when the activity is demanding. Such insights are valuable for refining the balance between system effectiveness and user comfort in future iterations. The TAM and I-UTAUT scales further confirmed participants’ confidence in using the platform and their recognition of its potential benefits for cognitive rehabilitation. Only the perceived system stability was statistically different among the two groups, as the healthy participants were slightly more critical than professionals. This finding could likely be due to different technological familiarity. Taken together, these findings suggest that PRoBio was well received across different user profiles, indicating good application of its design and interface.

The correlation analyses provided additional insight into usability, user experience, acceptance, and perceived workload of the PRoBio platform. The significant associations between HUBBI dimensions and I-UTAUT constructs (e.g., trust, social influence, overall acceptance) highlighted that technical reliability and interface quality are strong drivers of user’s confidence and intention to adopt the system. Similarly, the positive correlations between HUBBI and UEQ-S pragmatic quality indicated that a well-designed and functionally coherent interface enhances both perceived usefulness and emotional engagement. The negative associations observed between HUBBI or UEQ-S scores and NASA-TLX measures (e.g., frustration and physical demand) suggested that better usability and user’s experience are linked to lower cognitive and physical workload, confirming the non-burdensome nature of PRoBio. Interestingly, the TAM scales showed a positive relationship with NASA-TLX physical demand and overall workload, suggesting that more demanding interactions, particularly those involving the robotic arm, are perceived as beneficial for cognitive training. This finding is consistent with theories that moderate challenge can improve engagement and motivation. Based on these results, the Living Lab phase allowed us to detect these nuanced reactions and behaviour and identify both positive drivers (e.g., trust, pragmatic quality) and potential barriers (e.g., physical effort), providing actionable insights for further refinement of the PRoBio interface and functionalities prior to clinical implementation.

These results align with the growing literature supporting the use of Living Labs as effective methodologies for user-centered innovation in healthcare [33]. Previous studies have shown that Living Labs allow early detection of usability barriers, enhance user engagement, and promote the final refinement of complex systems prior to clinical deployment [34,35]. Defined as real-life environments that foster co-creation and iterative feedback, Living Labs are increasingly applied in digital health to bridge the gap between technological development and clinical applicability [33]. Moreover, combining Living Lab data with qualitative findings from the focus group offered a deeper understanding of both objective performance and subjective experience, providing a holistic view of the platform’s readiness for clinical application.

When compared to the current literature, PRoBio shows both innovative strengths and areas requiring further validation. Orgeta et al. [8] reported that cognitive training may benefit individuals with PD-MCI, but most studies lacked VR integration and did not systematically target higher-order cognitive domains such as executive functions or prospective memory. Additionally, motivational aspects and long-term engagement were often overlooked. Kwon et al. [9] found that VR-based interventions improved balance in PD, but evidence for broader functional outcomes was inconsistent, highlighting protocol variability and a lack of standardized follow-up.

PRoBio addresses some of these gaps by combining non-immersive VR with real-time feedback and bio-cooperative monitoring in a user-centered framework that targets multiple cognitive domains. These features may enhance engagement, motivation, and personalization. The emotional adaptability and trust elicited by PRoBio are promising indicators for its future clinical adoption. To support translational relevance, the platform is designed to be modular and compatible with existing rehabilitation workflows. For instance, PRoBio could be integrated into physiotherapy sessions by offering task-oriented motor exercises through the VRRS module, while the robotic arm supports interaction and feedback. Similarly, cognitive training programs could incorporate PRoBio’s virtual scenarios to target executive functions, attention, and memory, with real-time adaptation based on patient engagement and psychophysiological monitoring. Its flexibility allows clinicians to tailor sessions to individual needs, making it suitable for both in and outpatients. However, confirming Probio usability and acceptability (and its efficacy on cognitive outcomes) in real-world clinical settings will be essential before broader implementation. Successful deployment of PRoBio in clinical environments would require specific infrastructure and expertise. In hospital settings, integration would benefit from existing rehabilitation spaces equipped with robotic and digital therapy tools, as well as trained physiotherapists and neuropsychologists familiar with technology-assisted interventions. Staff would need brief training sessions to operate the platform, interpret feedback, and adapt exercises to patient needs.

The present two studies showed some limitations. First, a potential issue concerns the recruitment context, as participants were enrolled during a public Parkinson’s Disease event. This may have introduced a selection bias, favoring the inclusion of individuals, both patients and professionals, who are particularly motivated, engaged, or already active in disease management or advocacy. As a result, the generalizability of the findings to the broader Parkinson’s population, especially those less inclined to participate in such initiatives, may be limited. However, the high level of engagement of these participants allowed for rich, meaningful feedback that is valuable for guiding early-stage design and development of the PRoBio platform. Second, although the session was described as a focus group, it did not fully align with the methodological features of a traditional, moderated group discussion. The procedure more closely resembled a group-administered survey, followed by a spontaneous and informal exchange with participants. This hybrid format nonetheless allowed the collection of both quantitative and qualitative data. Importantly, the qualitative feedback that emerged during the informal discussion was consistent and thematically coherent, supporting the validity of the insights obtained despite the methodological deviation.

Moreover, the Living Lab involved a relatively small sample of participants, including only six healthy volunteers and eight healthcare professionals. While this limits the generalizability of the findings to broader clinical populations, the choice was deliberate and aligned with the Living Lab methodology, where formal sample size calculation is not mandatory [20]. A further key limitation is the absence of participants from the target population (i.e., patients with PD-MCI). Although the inclusion of healthy volunteers and healthcare professionals allowed for a controlled and safe evaluation of system usability and workflow integration, their experience cannot fully capture the challenges faced by patients with motor and cognitive impairments. This methodological choice was intentional, as the Living Lab served as a preparatory phase prior to clinical testing. Nonetheless, the lack of direct patient input in this phase reduces ecological validity and should be considered when interpreting the results. To address this, subsequent phases of the OPERA project include a pilot study with PD-MCI patients, which will directly evaluate usability, feasibility, and clinical applicability in the intended population. In addition, the study lacked a longitudinal or follow-up component, precluding any conclusions about sustained engagement, long-term usability, or single session-training efficacy. Furthermore, the robustness of the PRoBio integration of physiological monitoring and adaptive feedback has yet to be systematically evaluated. Finally, although the difference in SUS scores between healthcare professionals (70.1) and healthy volunteers (83.0) did not reach statistical significance, it remains noteworthy from a practical perspective. Both scores reflect acceptable to excellent usability. However, the lower rating from professionals may stem from a more critical viewpoint, shaped by clinical experience, prior exposure to rehabilitation technologies, and higher expectations for system reliability and integration into care workflows. In contrast, healthy volunteers, less familiar with such technologies, reported a more positive experience. This divergence provides valuable insight, underscoring the need for future design refinements that meet clinical standards, while preserving the accessibility and user-friendliness appreciated by non-professional users. These considerations are consistent with broader discussions in the Living Lab literature, which often emphasize challenges related to sustainability, scalability, and standardization [36].

## 5. Conclusions

Notwithstanding these limitations, this two-part study allowed us to identify both strengths and potential barriers of the PRoBio system and its core components, providing valuable insights to guide its refinement prior to clinical implementation. The preliminary findings suggested that the design choices implemented in the platform are promising in terms of usability and acceptability. While Study 1 and Study 2 involved different participants and methodologies, and no direct causal link can be drawn, the positive outcomes observed in the Living Lab indicate that the early feedback collected in Study 1 was broadly aligned with user needs. This coherence highlights the value of integrating multi-stakeholder input during the early stages of development, especially in user-centred neurorehabilitation technologies.

Further validation with individuals with PD-MCI is essential to ensure the platform’s usability and relevance to their specific cognitive and functional profiles. A pilot study already planned within the OPERA project will evaluate treatment adherence, usability, and preliminary clinical effects. However, a randomized controlled trial on a larger patient cohort will ultimately be needed, to establish its clinical effectiveness and inform its integration into routine, personalized rehabilitation pathways.

## Figures and Tables

**Table 1:** Distribution of patients, caregivers and health professionals’ responses to the targeted questionnaires.

Section	Question	Response from Patients (% of Respondents)	Response from Caregiver (% of Respondents)	Response from Healthcare Professionals (% of Respondents)
Section 2—Clinical-Functional Aspects	Daily difficulties *	-Following a long or complex conversation (50.0%) -Get oriented (30.0%) -Remembering meetings (10.0%) -Other (10.0%)	-Following a long or complex conversation (40.0%) -Get oriented (40.0%) -Attention-demanding tasks (20.0%)	-Following a long or complex conversation (37.5%) -Attention-demanding tasks (37.5%) -Remembering meeting (12.5%) -Other (12.5%)
	Frequency of cognitive activities	-Every day (60.0%) -Some days (10.0%) -Rarely (30.0%)	-Every day (20.0%) -Some days (60.0%) -Rarely (20.0%)	-
	Importance of cognitive abilities	-Very important (90.0%) -Important (10.0%)	-Very important (60.0%) -Important (20.0%) -Slightly important (20.0%)	-
Section 3—Technical Expectations and Preferences	Key aspects of system (robot/VR) **	-Ease of use (60.0%) -Personalization (30.0%) -Engagement (50.0%) -Progress monitoring (10.0%)	-Ease of use (100.0%) -Safety (40.0%) -Personalization (40.0%) -Engagement (80.0%) -Progress monitoring (60.0%)	-Ease of use (50.0%) -Personalization (75.5%) -Progress monitoring (62.5%)
	Preferred activities/exercises (robot/VR) **	-Memory games (50.0%) -Virtual simulations (20.0%) -Cognitive challenges (40.0%)	-Memory games (80.0%) -Virtual simulations (40.0%) -Cognitive challenges (60.0%)	-Memory games (12.5%) -Virtual simulations (75.0%) -Cognitive challenges (37.5%)
	Importance of immediate feedback	-Very important (60.0%) -Important (40.0%)	-Very important (100.0%)	-Very important (62.5%) -Important (37.5%)
	Main goals of cognitive rehabilitation *	-Autonomy in daily activities (50.0%) -Reduce anxiety and stress (30.0%) -Improve memory (10.0%) -Increase concentration (10.0%)	-Autonomy in daily activities (40.0%) -Reduce anxiety and stress (40.0%) -Improve memory (20.0%)	-Autonomy in daily activities (75.0%) -Reduce anxiety and stress (12.5%) -Improve memory (12.5%)
Section 4—General Health and Mobility	Motor difficulties (impact tech use)	-Yes (20.0%)	-Yes (40.0%)	-Yes (50.0%)
	Fine motor difficulties	-Yes (60.0%)	-Yes (80.0%)	-Yes (87.5%)
	Autonomous use of technology devices	-Yes (70.0%)	-Yes (100.0%)	-Yes (87.5%)
	Physical needs/ limitations for design	-Yes (20.0%)	-Yes (40.0%)	-Yes (25%)
Section 5—Robotic Interaction and Expectations	Useful support by robotic arm *	-Cognitive activities (60.0%) -Physical movements (40.0%)	-Cognitive activities (80.0%) -Physical movements (20.0%)	-Cognitive activities (62.5%) -Physical movements (37.5%)
	Preferred activities with robotic arm **	-Manipulating objects (20.0%) -Simulating daily activities (20.0.7%) -Motor Rehabilitation (40.0%)	-Manipulate objects (20.0%) -Complete Puzzles (40.0%) -Simulate daily activities (40.0%) -Motor Rehabilitation (40.0%)	-Manipulate objects (25.0%) -Complete puzzles (37.5%) -Simulate daily activities (87.5%) -Motor Rehabilitation (62.5%)
	Desired technical features (robotic arm) *	-Adjusting arm gestures (30.0%) -Soft and precise movements (30.0%) -Safety in use (20.0%) -Other (20.0%)	-Adjusting arm gestures (20.0%) -Soft and precise movements (20.0%) -Safety in use (20.0%) -Other (40.0%)	-Adjusting arm gestures (62.5%) -Soft and precise movements (12.5%) -Safety in use (25.0%)
	Importance of robot detecting emotional/ fatigue states *	-Very important (50.0%) -Important (50.0%)	-Very important (80.0%) -Important (20.0%)	-Very important (87.5%) -Important (12.5%)

Legend: HP = Healthcare Professionals; * Single answer; ** Multiple answer.

**Table 2:** Questionnaire scores and statistical comparisons between healthy volunteers and healthcare professionals.

Questionnaire/Subscale	Healthcare Professionals (Mean; SD)	Healthy Volunteers (Mean; SD)	*p*-Value
SUS (Total)	70.1 (12.3)	83.0 (10.5)	0.23
HUBBI—Basic System Performance	3.5 (0.8)	4.2 (0.7)	0.04
HUBBI—Task-Technology Fit	3.8 (0.7)	4.0 (0.6)	0.13
HUBBI—Design and Presentation	3.9 (0.9)	4.2 (0.6)	0.26
HUBBI—Navigation and Structure	4.0 (0.7)	4.1 (0.5)	0.66
HUBBI—Information and Terminology	3.7 (0.8)	3.8 (0.5)	0.65
HUBBI—Guidance and Support	3.6 (0.6)	3.8 (0.4)	0.31
HUBBI—Satisfaction	4.1 (0.7)	4.2 (0.5)	0.58
UEQ-S—Pragmatic Quality	1.1 (0.4)	1.1 (0.5)	0.94
UEQ-S—Hedonic Quality	0.9 (0.5)	1.2 (0.4)	0.61
UEQ-S (Total)	1.0 (0.4)	1.2 (0.4)	0.79
TAM—Perceived Usefulness (PU)	5.2 (1.1)	5.4 (0.9)	0.63
TAM—Perceived Ease of Use (PEOU)	5.0 (1.2)	5.2 (0.8)	0.48
I-UTAUT—Performance Expectancy	3.8 (0.9)	3.9 (0.8)	0.96
I-UTAUT—Social Influence	3.6 (0.8)	3.7 (0.7)	0.90
I-UTAUT—Facilitating Conditions	3.9 (0.9)	4.0 (0.8)	0.84
I-UTAUT—Self-Efficacy	3.7 (0.7)	3.8 (0.6)	0.91
I-UTAUT—Anxiety	2.8 (0.6)	2.9 (0.5)	0.48
I-UTAUT (Total)	3.7 (0.8)	3.8 (0.7)	0.90
NASA-TLX—Mental Demand	30.2 (8.5)	28.5 (7.9)	0.65
NASA-TLX—Physical Demand	25.1 (6.7)	24.3 (5.9)	0.56
NASA-TLX—Temporal Demand	28.3 (7.4)	29.1 (7.1)	0.27
NASA-TLX—Performance	75.0 (9.2)	77.3 (8.5)	0.41
NASA-TLX—Effort	35.1 (8.1)	34.3 (7.5)	0.72
NASA-TLX—Frustration	20.0 (7.3)	21.1 (6.9)	0.84
NASA-TLX (Total)	35.1 (6.8)	35.6 (6.5)	0.90

Legend. HUBBI = eHealth UsaBility Benchmarking Instrument; I-UTAUT = Italian version of the Unified Theory of Acceptance and Use of Technology; NASA-TLX = NASA Task Load Index; SUS = System Usability Scale; TAM = Technology Acceptance Model; UEQ-S = User Experience Questionnaire—Short version.

## Data Availability

The data presented in this study are available on reasonable request from the corresponding author. The data are not publicly available due to privacy and ethical restrictions.

## Supplementary Materials

The following supporting information can be downloaded at: https://www.mdpi.com/article/10.3390/jcm14197042/s1, Section S1: Questionnaires administered to patients, caregivers, and healthcare professionals during the Focus Group; Section S2: Detailed description of the Living Lab questionnaires: System Usability Scale (SUS), eHealth Usability Benchmarking Instrument (HUBBI), User Experience Questionnaire—Short version (UEQ-S), Technology Acceptance Model (TAM), Italian version of the Unified Theory of Acceptance and Use of Technology (I-UTAUT), and NASA Task Load Index (NASA-TLX).

## Abbreviations

The following abbreviations are used in this manuscript:

GSR	Galvanic Skin Response
HUBBI	eHealth UsaBility Benchmarking Instrument
I-UTAUT	The Italian version of the Unified Theory of Acceptance and Use of Technology
MCI	Mild Cognitive Impairment
NASA-TLX	NASA Task Load Index
PD	Parkinson’s Disease
PRoBio	Bio-Cooperative Robotic Platform
RGB-D	Red Green Blue-Depth camera
SUS	System Usability Scale
TAM	Technology Acceptance Model scales
UCBM	Campus Bio-Medico University of Rome
UEQ-S	Short Version of the User Experience Questionnaire
VR	Virtual Reality
VRRS	Virtual Reality Rehabilitation System
