# Supplementary material for: Preliminary User-Centred Evaluation of a Bio-Cooperative Robotic Platform for Cognitive Rehabilitation in Parkinson’s Disease and Mild Cognitive Impairment: Insights from a Focus Group and Living Lab in the OPERA Project
Source: J Clin Med. 2025 Oct 5;14(19):7042. doi: 10.3390/jcm14197042 (PMC12525349; doi:10.3390/jcm14197042)
Supplement: Supplementary file 1 [file jcm-14-07042-s001.zip › jcm-3845637-supplementary.pdf]

Supplementary materials

Section S1

Here, we present the questionnaires administered to patients, caregivers, and healthcare professionals during the Focus Group.

Questionnaire for the design of a new Bio-Cooperative Platform for the cognitive rehabilitation of patients with Parkinson's Disease (PD)

OPERA Project

Patient Version

Dear Participant,

Thank you for taking part in this study.

Your responses to this questionnaire will help us to understand your experience of, expectations of, and opinions on the use of robotic technologies for cognitive rehabilitation.

The goal is to gather insights from people like you who are living with Parkinson's Disease, so that we can design a tool that meets real needs. The responses will be used for research purposes to help develop an innovative system for cognitive rehabilitation.

Completing the questionnaire should take around 10 minutes.

All responses will be handled anonymously and kept strictly confidential.

SECTION 1: Demographic

1. **Age**: _________ years old
2. **Gender**: ☐ Male ☐ Female ☐ Other
3. **How long ago were you diagnosed with Parkinson's Disease?**
4. ☐ Less than 1 year
   ☐ 1-3 years
   ☐ 4-6 years
   ☐ More than 6 years
5. **Which treatments are you currently undergoing?**
   ☐ Medication ☐ Physiotherapy ☐ Speech therapy ☐ Other: ___________
6. **Have you ever used technology such as robots or virtual reality for rehabilitation?**
   ☐ Yes ☐ No
   Please specify: _______________

SECTION 2: Clinical-Functional information

7. **Which everyday activity is the most challenging for you?** *(single choice)*

☐ Remembering appointments

☐ Finding his way around unfamiliar places

☐ Following long or complex conversations

☐ Staying focused on tasks that require sustained attention

☐ Other (please specify): ___________

8. **How often do you take part in activities that stimulate cognitive abilities (e.g., puzzles, reading)?**

☐ Daily ☐ Several times a week ☐ Rarely ☐ Never

9. **How important is it for your family member to maintain and improve their cognitive skills?**

☐ Very important ☐ Important ☐ Not very important ☐ Not important at all

SECTION 3: Technical Expectations and Preferences

10. **Which features do you consider are most important for a rehabilitation system using robots or virtual reality?** *(multiple choice)*

☐ Easy to use

☐ Safe

☐ Customisation

☐ Engaging and enjoyable

☐ Progress monitoring

☐ Other (please specify): ___________

11. **What activities or exercises would you include in a rehabilitation programme involving robots and/or virtual reality?** *(multiple choice)*

☐ Memory games (e.g., word lists, remembering object locations)

☐ Virtual simulations of everyday tasks (e.g., shopping, cooking)

☐ Cognitive tasks (e.g., puzzles or logic games)

☐ Other (please specify): __________

12. **How important is it that the system gives you immediate feedback on your progress?**

☐ Very important

☐ Important

☐ Slightly important

☐ Not important

13. **Which goal do you think is the most important for you to achieve through cognitive rehabilitation?** *(single choice)*

☐ Improving memory

☐ Enhancing focus and attention

☐ Reducing anxiety or stress

☐ Gaining more independence in daily life

☐ Other (please specify): __________

SECTION 4: General Health and Mobility

14. **Do you have movement difficulties that might make it harder to use technology?**

☐ Yes ☐ No

Please specify: __________

15. **Have you ever struggled with fine motor activities (e.g., using a mouse or smartphone, or writing)?**

☐ Yes ☐ No

Please specify: __________

16. **Are you able to use digital devices (e.g., smartphone or tablet) on your own?**

☐ Yes ☐ No

17. **Do you have any special physical needs or limitations we should consider when designing the system?**

☐ Yes ☐ No
Please specify: __________

<u>SECTION 5</u>: Robotic Interaction and Expectations

18. **Have you ever used robotic devices for physical or cognitive rehabilitation?**
☐ Yes ☐ No

Which ones: __________

19. **What kind of help would you find most useful from a robotic arm during rehabilitation?** *(single choice)*
☐ Assistance with physical movement (e.g., guiding the arm or hand)

☐ Support during cognitive tasks (e.g., task simulations)

☐ Other (please specify): __________

20. **Which activities do you think you like to perform with the robotic arm?** *(multiple choice)*
☐ Moving and handling objects
☐ Completing puzzles or building games

☐ Simulating everyday tasks (e.g., cooking, writing)
☐ Performing physical therapy exercises (e.g., guided, repetitive motions)
☐ Other (please specify): __________

21. **Which technical features would you like the robotic arm to have?** *(single choice)*
☐ Ability to perform delicate and precise movements

☐ Ability to adapt its movements to the user's current physical abilities

☐ Safety of use (e.g., adjusting strength to avoid resistance or injury)

☐ Other (please specify): __________

22. **Do you have any concerns about using a robotic arm?** *(single choice)*
☐ Feeling "controlled" by the robot

☐ Difficulty adapting to the robot's movement

☐ Worry that the system might be too complicated

☐ Other (please specify): __________

23. **How important is it to you that the robot can recognize your emotional state and fatigue during use?** *(single choice)*

☐ Very important

☐ Important

☐ Slightly important

☐ Not important

SECTION 6: Suggestions and Open Feedback

24. **What do you think the main advantages of a rehabilitation system using robots and/or virtual reality could be?**

25. **Do you have any concerns about what might make using such a system difficult?**

Caregiver Version

Dear Participant,

Thank you for taking part in this study.

Your responses to this questionnaire will help us to understand your experience of, expectations of, and opinions on the use of robotic technologies for cognitive rehabilitation.

Our aim is to gather valuable insights from individuals who care for people with Parkinson's disease. Your insights will inform the development of an innovative tool to support cognitive rehabilitation. Your contribution will help to create a system that meets the needs of patients and carers.

Completing the questionnaire should take around 10 minutes.

All responses will be handled anonymously and kept strictly confidential.

SECTION 1: Demographic

1. **Age**: _________ years old
2. **Gender**: ☐ Male ☐ Female ☐ Other
3. **Relationship:**

☐ Spouse ☐ Parent ☐ Sibling ☐ Professional Caregiver ☐ Other:_______

4. **When was your family member diagnosed with Parkinson's disease?**

☐ Less than 1 year
☐ 1-3 years
☐ 4-6 years
☐ More than 6 years

5. **Which treatments is your family member currently undergoing?**

☐ Medication ☐ Physiotherapy ☐ Speech therapy ☐ Other: __________

6. **How long have you been caring for your family member with Parkinson's disease?**

☐ Less than 1 year
☐ 1-3 years
☐ 4-6 years
☐ More than 6 years

7. **Has your family member ever used technology such as robots or virtual reality for rehabilitation?**

☐ Yes ☐ No
Please specify: _______________

SEZIONE 2: Clinical-Functional information

8. **Which everyday activity is the most challenging for your family member?** *(single choice)*

☐ Remembering appointments

☐ Finding his way around unfamiliar places

☐ Following long or complex conversations

☐ Staying focused on tasks that require sustained attention
☐ Other (please specify): __________

9. **How often does your family member take part in activities that stimulate cognitive abilities (e.g., puzzles, reading)?**

☐ Daily ☐ Several times a week ☐ Rarely ☐ Never

10. **How important is it for your family member to maintain and improve their cognitive skills?**

☐ Very important ☐ Important ☐ Not very important ☐ Not important at all

SECTION 3: Technical Expectations and Preferences

11. **Which features are most important for a rehabilitation system using robots or virtual reality for your family member?** *(multiple choice)*

☐ Easy to use

☐ Safe

☐ Customisation

☐ Engaging and enjoyable

☐ Progress monitoring

☐ Other (please specify): ___________

12. **What activities or exercises would you include for your family member in a rehabilitation programme involving robots and/or virtual reality?** *(multiple choice)*

☐ Memory games (e.g., word lists, remembering object locations)

☐ Virtual simulations of everyday tasks (e.g., shopping, cooking)

☐ Cognitive tasks (e.g., puzzles or logic games)

☐ Other (please specify): ___________

13. **How important is it to you that the system provides your family member with immediate feedback on his progress?**

☐ Very important

☐ Important

☐ Slightly important

☐ Not important

14. **Which goal do you think is the most important for your family member to achieve through cognitive rehabilitation?** *(single choice)*

☐ Improving memory

☐ Enhancing focus and attention

☐ Reducing anxiety or stress

☐ Gaining more independence in daily life

☐ Other (please specify): __________

SECTION 4: General Health and Mobility

15. **Could your family member's motor difficulties affect his ability to use technological devices?**

☐ Yes ☐ No

Please specify: __________

16. **Has your family member ever struggled with fine motor activities (e.g., using a mouse or smartphone, or writing)?**

☐ Yes ☐ No

Please specify: __________

17. **Can your family member use tech devices (e.g., smartphone or tablet) on his own?**

☐ Yes ☐ No

18. **Are there any specific physical needs or limitations we should take into account when designing the system?**

☐ Yes ☐ No

Please specify: __________

SECTION 5: Robotic Interaction and Expectations

19. **Has your family member ever used robotic devices for physical or cognitive rehabilitation?**

☐ Yes ☐ No

Which ones: __________

20. **Which type of support from a robotic arm would you find most useful when helping your family member with their rehabilitation?** *(single choice)*

☐ Assistance with physical movement (e.g., guiding the arm or hand)

☐ Support during cognitive tasks (e.g., task simulations)

☐ Other (please specify): __________

21. **Which activities do you think your family member could perform with the robotic arm?** *(multiple choice)*

☐ Moving and handling objects

☐ Completing puzzles or building games

☐ Simulating everyday tasks (e.g., cooking, writing)

☐ Performing physical therapy exercises (e.g., guided, repetitive motions)

☐ Other (please specify): __________

22. **Which technical features would you like the robotic arm to have for your family member?** *(single choice)*

☐ Ability to perform delicate and precise movements

☐ Ability to adapt its movements to the user's current physical abilities

☐ Safety of use (e.g., adjusting strength to avoid resistance or injury)

☐ Other (please specify): __________

23. **What are the potential concerns that your family member might have regarding the use of a robotic arm?** *(single choice)*

☐ Feeling "controlled" by the robot

☐ Difficulty adapting to the robot's movement

☐ Worry that the system might be too complicated

☐ Other (please specify): __________

24. **How important is it to you that the robot can recognize your family member's emotional state and fatigue during use?** *(single choice)*

☐ Very important

☐ Important

☐ Slightly important

☐ Not important

SECTION 6: Suggestions and Open Feedback

25. **What do you think the main advantages of a rehabilitation system using robots and/or virtual reality could be?**

---

26. **Do you have any concerns about what might make using such a system difficult?**

Healthcare Professional Version

Dear Participant,

Thank you for taking part in this study.

Your responses to this questionnaire will help us to understand your experience of, expectations of, and opinions on the use of robotic technologies for cognitive rehabilitation.

Our aim is to gather valuable insights from individuals who care for people with Parkinson's disease. Your insights will inform the development of an innovative tool to support cognitive rehabilitation. Your contribution will help to create a system that meets the needs of patients and carers.

Completing the questionnaire should take around 10 minutes.

All responses will be handled anonymously and kept strictly confidential.

SECTION 1: Demographic

1. **Age**: _________ years old
2. **Gender**: ☐ Male ☐ Female ☐ Other
3. **Profession:**

☐ Medical Doctor ☐ Physiotherapist ☐ Psychologist ☐ Speech Therapist

☐ Occupational Therapist ☐ Nurse ☐ Healthcare Worker

☐ Other: ____________________

4. **Years of experience as a healthcare professional:**

☐ Less than 1 year

☐ 1–3 years

☐ 4–6 years

☐ More than 6 years

5. **Years of experience with patients with Parkinson's Disease:**

☐ None

☐ Less than 1 year

☐ 1–3 years

☐ 4–6 years

☐ More than 6 years

SECTION 2: Clinical-Functional information

(Complete this section only if you have experience with PD patients)

6. **Which cognitive domain do you find most impaired in PD patients?** *(single choice)*

☐ Memory

☐ Attention and concentration

☐ Planning and organizational skills

☐ Spatial and temporal orientation

☐ Other (please specify): ___________

7. **Which everyday activity is the most challenging for these patients?** *(single choice)*

☐ Remembering appointments

☐ Finding his way around unfamiliar places

☐ Following long or complex conversations

☐ Staying focused on tasks that require sustained attention

☐ Other (please specify): ___________

SECTION 3: Technical Expectations and Preferences

8. **Which features are most important for a rehabilitation system using robots or virtual reality for PD patients?** *(multiple choice)*

☐ Easy to use

☐ Safe

☐ Customisation

☐ Engaging and enjoyable

☐ Progress monitoring

☐ Other (please specify): __________

9. **What activities or exercises would you include for these patients in a rehabilitation programme involving robots and/or virtual reality?** *(multiple choice)*

☐ Memory games (e.g., word lists, remembering object locations)

☐ Virtual simulations of everyday tasks (e.g., shopping, cooking)

☐ Cognitive tasks (e.g., puzzles or logic games)

☐ Other (please specify): __________

10. **How important is it to you that the system provides these patients with immediate feedback on his progress?**

☐ Very important

☐ Important

☐ Slightly important

☐ Not important

11. **Which goal do you think is the most important for PD patients to achieve through cognitive rehabilitation?** *(single choice)*

☐ Improving memory

☐ Enhancing focus and attention

☐ Reducing anxiety or stress

☐ Gaining more independence in daily life

☐ Other (please specify): __________

SECTION 4: General Health and Mobility

(Complete this section only if you have experience with PD patients)

12. **Could PD patients motor difficulties affect their ability to use technological devices?**

☐ Yes ☐ No

Please specify: __________

13. **Have you observed difficulties with fine motor tasks (e.g., using a mouse, smartphone, or writing)?**

☐ Yes ☐ No

Please specify: __________

14. **Are patients generally able to use technological devices (e.g., smartphone, tablet) on their own?**

☐ Yes ☐ No

15. **Are there any specific physical needs or limitations we should take into account when designing the system?**

☐ Yes ☐ No

Please specify: __________

SECTION 5: Robotic Interaction and Expectations

16. **Have you ever used robotic devices for the physical or cognitive rehabilitation of patients with neurological conditions?**

☐ Yes ☐ No

Which ones: __________

17. **What type of support do you think would be most useful from a robotic arm in neurological rehabilitation?** *(single choice)*

☐ Assistance with physical movement (e.g., guiding the arm or hand)

☐ Support during cognitive tasks (e.g., task simulations)

☐ Other (please specify): __________

18. **Which activities do you think neurological patients could perform with the robotic arm?** *(single choice)*

☐Moving and handling objects

☐ Completing puzzles or building games

☐ Simulating everyday tasks (e.g., cooking, writing)

☐ Performing physical therapy exercises (e.g., guided, repetitive motions)

☐ Other (please specify): ___________

19. **Which technical features would you like the robotic arm to have?** *(single choice)*

☐ Ability to perform delicate and precise movements

☐ Ability to adapt its movements to the user's current physical abilities

☐ Safety of use (e.g., adjusting strength to avoid resistance or injury)

☐ Other (please specify): ___________

20. **What are the potential concerns that PD patients might have regarding the use of a robotic arm?** *(single choice)*

☐ Feeling "controlled" by the robot

☐ Difficulty adapting to the robot's movement

☐ Worry that the system might be too complicated

☐ Other (please specify): ___________

21. **How important is it that the robot can detect a patient's emotional state and fatigue during exercises?** *(single choice)*

☐ Very important

☐ Important

☐ Slightly important

☐ Not important

<u>SECTION 6</u>: Suggestions and Open Feedback

22. **What do you think the main advantages of a rehabilitation system using robots and/or virtual reality could be?**

---

23. Do you have any concerns about what might make using such a system difficult?

Section S2

Here it is provided a detailed description of the Living Lab questionnaires:

System Usability Scale (SUS; Brooke, 1996; Lewis et al., 2018)

The SUS is a self-report questionnaire composed of 10 items designed to measure perceived system usability. It evaluates key aspects of user experience, including ease of use (assessing the user's perception of the overall ease of using the system), familiarity and consistency (the extent to which the system appears intuitive or familiar), and overall satisfaction (the degree of acceptance and enjoyment derived from interaction with the system).

Participants respond to each item using a 5-point Likert scale, where 1 means “Strongly disagree” and 5 means “Strongly agree.” Items alternate between positively and negatively worded statements to reduce response bias. For positive items (odd-numbered), the participant’s score is reduced by 1; for negative items (even-numbered), the score is subtracted by 5. The adjusted scores are summed and multiplied by 2.5, yielding a final score ranging from 0 to 100, where higher scores indicate better perceived usability.

eHealth Usability Benchmarking Instrument (HUBBI; Broekhuis et al., 2023)

The Hubbi is a self-report questionnaire developed specifically to evaluate the usability of eHealth services. The questionnaire consists of 18 items distributed across seven dimensions, each reflecting a critical aspect of interaction between users and eHealth systems: Basic System Performance, used to assess the speed, reliability, and technical stability of the system; Task-Technology Fit; for the measures of how well the system supports users in completing relevant tasks; Design & Presentation, that evaluates the aesthetic quality and visual clarity of the interface; Navigation & Structure concerns the ease with which users can find their way through the system and understand its organizational layout; Information & Terminology, related to the clarity, accuracy, and comprehensibility of the content and language used; Guidance & Support, focuses on the availability and helpfulness of support resources such as help features or tutorials; and Satisfaction, to capture the user's overall satisfaction with the system experience.

Each item is rated on a 5-point Likert scale, where 1 corresponds to "Strongly disagree" and 5 to "Strongly agree." The scoring procedure involves computing the mean score for each dimension as well as an overall average usability score across all items. Higher scores indicate better perceived usability.

User Experience Questionnaire – Short version (UEQ-S; Schrepp et al., 2017)

The UEQ-S is a brief self-report questionnaire consisting of 8 items designed to rapidly measure the user experience of a system. Items are divided into two main factors: Pragmatic Quality (PQ), for the assessment of the system's effectiveness, efficiency, and ease of use; and Hedonic Quality (HQ), which captures enjoyment, emotional engagement, and aesthetic appeal.

Each item is presented as a semantic differential, using bipolar adjective pairs (e.g., "complicated–easy", "boring–exciting"). Participants are asked to rate their experience on a 7-point Likert scale ranging from -3 to +3, where -3 indicates full agreement with the negative term, +3 with the positive term, and 0 represents a neutral position. For each factor, the arithmetic mean of the corresponding item scores is calculated. Higher scores in PQ indicate better usability, while higher scores in HQ reflect greater emotional satisfaction and engagement.

Technology Acceptance Model scales (TAM; Davis, 1989; Venkatesh & Bala, 2008*)*

The TAM is a self-report instrument designed to measure the determinants of user acceptance of information technology. Grounded in the Theory of Reasoned Action (TRA), TAM aims to explain how users come to accept and use a technology, focusing on two core cognitive beliefs that influence behavioural intention and, ultimately, actual system use. The questionnaire consists of 12 items, divided into two primary constructs: Perceived Usefulness (PU), referring to the degree to which an individual believes that using a specific system will enhance cognitive rehabilitation. It captures the instrumental value of the technology in facilitating task completion and achieving desired outcomes; Perceived Ease of Use (PEOU), used to evaluate the degree to which an individual believes that using the system will be free of effort. It reflects the user's perception of the system's usability and cognitive simplicity.

Each construct is measured using six items, and responses are collected using a 7-point Likert scale, ranging from "Strongly disagree" (1) to "Strongly agree" (7). Scoring involves calculating the mean score for each of the two dimensions separately. Higher scores in PU suggest a stronger belief in the system's practical benefits, while higher scores in PEOU indicate that the system is perceived as user-friendly and easy to interact with.

Italian version of the Unified Theory of Acceptance and Use of Technology (I-UTAUT; D'Iorio et al., 2025)

The I-UTAUT is a self-report questionnaire developed to assess technology acceptance. It is composed of 41 items, organized into twelve constructs that reflect the multidimensional nature of technology acceptance: Performance Expectancy, the belief that using the system will improve performance; Effort Expectancy, the perceived ease of using the system; Attitude Toward Using Technology, the overall affective evaluation of using the system; Social Influence, the degree to which individuals perceive that others believe they should use the system; Facilitating Conditions, the perceived availability of support and resources for system use; Self-Efficacy, users' confidence

in their own ability to use the system; Anxiety, the extent of apprehension or fear related to system use; Behavioural Intention to Use, the intention to continue using the system in the future.

Participants respond to each item using a 5-point Likert scale, ranging from 1 (“Strongly disagree”) to 5 (“Strongly agree”). Scoring is conducted by computing the mean score for each individual construct. Higher scores indicate more favourable perceptions of that particular factor.

NASA Task Load Index (NASA-TLX; Hart & Staveland, 1988; Devos et al., 2020*)*

The NASA-TLX is a self-report tool designed to measure an individual’s perceived workload during task execution. The NASA-TLX consists of six dimensions, each capturing a specific aspect (GSR) of subjective workload: Mental Demand, the amount of mental and perceptual activity required (e.g., thinking, deciding, calculating); Physical Demand, the amount of physical effort required to complete the PRoBio system; Temporal Demand, the time pressure felt due to PRoBio system pace and urgency; Performance, the user’s self-assessment of PRoBio system success and satisfaction with the outcome; Effort, the degree of mental and physical work exerted to reach the performance level; Frustration Level, the extent of negative emotional response, such as irritation, stress, or annoyance. Each of these six factors is rated on a 0–100 continuous scale (visual analog slider) anchored at 0 (“Very low”) and 100 (“Very high”). Tick marks at 5-point intervals may be shown only as visual guides; participants can select any value.

Each subscale is rated independently based on these comparisons, and weights are assigned to each dimension (ranging from 0 to 5), which are then used to compute a weighted average workload score. Alternatively, a Raw TLX score can be calculated by averaging the unweighted ratings across all six dimensions, offering a quicker but less tailored measure.
